# Associations Between Sleep Patterns and Performance Development Among Norwegian Chess Players

**DOI:** 10.3389/fpsyg.2020.01855

**Published:** 2020-07-28

**Authors:** Frode Moen, Maja Olsen, Maria Hrozanova

**Affiliations:** ^1^Department of Education and Lifelong Learning, Faculty of Social and Educational Sciences, Norwegian University of Science and Technology, Trondheim, Norway; ^2^Centre for Elite Sports Research, Department of Neuromedicine and Movement Science, Faculty of Medicine and Health Science, Norwegian University of Science and Technology, Trondheim, Norway

**Keywords:** sleep, chess, performance, development, rating

## Abstract

An inherent part of elite-level chess are high emotional and cognitive stress loads related to performance development. Sleep is a crucial recovery strategy, previously implicated in athletic performance. The main purpose of the current study was to investigate the associations between performance development and objectively measured sleep in a sample of 14 Norwegian chess players over a period of 120 consecutive days. Seven of the chess players in the current sample had negative development in their International Chess Federation (FIDE) ranking score in the period of sleep monitoring, while 7 had positive development. The sleep patterns of the chess players with positive performance development were different from the players with negative development – with higher amounts of deep sleep, less rapid eye movement (REM) sleep and lower respiration rate in the positive performance development group compared to the negative performance development group. The findings are discussed in terms of existing knowledge on the importance of sleep stage distribution and sleep durations for athlete functioning, and in light of applied implications and possible future research.

## Introduction

Chess is a game described as “mental torture” for the player because of the involvement of the many complex cognitive processes, often over a long period of time (Kaya and Öztürk, 2015). Cognitive processes such as strategic thinking, deep concentration, constantly seeking in episodic memory and comparing chess positions with earlier experiences, involvement in problem solving, calculation and decision making, are all essential to reaching high performance levels in chess tournaments (Kaya and Öztürk, 2015). These cognitive abilities enable chess players to perceive the board pattern of own and opponent’s chess pieces positions, to predict the best move based on the memories of chess positions and experiences from games and trainings, and to make a quick generation of the best next move (De Groot and Gobet, 1996; Gobet and Waters, 2003). In this way, the chess player is under significant pressure to make the best possible use of the cognitive abilities underlying the chess performance (Fuentes-García et al., 2019a).

In addition to the pressures of performing on the spot, chess players are also faced with being constantly evaluated for their performances. Indeed, chess players are ranked on a worldwide ranking list by the International Chess Federation (FIDE) based on the matches they play. Thus, the players’ performance scores are always in focus, their every move on the chess board has direct consequences on the international FIDE rating scores, and their competitiveness is always visible on the FIDE’s web page. These challenges represent relevant and potent acute stressors for the players. Such circumstances will normally lead to the experience of stress – both during chess games (Kaya and Öztürk, 2015; Fuentes-García et al., 2019b) and after games because of a potential prolonged activation of the stress response (Brosschot et al., 2005). The prolonged activation of the stress response occurs when situational demands exceed players’ capabilities to meet demands over time, for instance if they lose game after game and their FIDE rating scores decrease (Schneiderman et al., 2005). In such cases, repeated or chronic engagement in cognitive activations such as worrying, rumination and cognitive arousals, together termed preservative cognitions, keeps the stress-related content from resolving. As a result, the stress response systems of the body remain activated, and the stress response continues also in the absence of acute stressors (Brosschot et al., 2005).

Chess players’ performances are undoubtably highly associated with their cognitive abilities such as attentional skills, strategic thinking, retrieval of relevant information from memory, and making the best possible move (Schneider et al., 1993; Gobet and Charness, 2006; Kaya and Öztürk, 2015; Burgoyne et al., 2016). Accordingly, during the experience of overwhelming stressors and preservative cognitions, players’ attention is disposed to be locked into persistent patterns of negative thinking that are difficult to control (Wells, 2009). Such style of thinking is defined as Cognitive Attentional Syndrome (CAS). CAS often contributes to negative emotions such as nervousness and anxiety (Wells, 2009). Thus, players’ abilities to self-regulate emotions during the stress response are also essential for deciding the outcome of the given stressful scenario (De Groot et al., 1996; Gobet and Waters, 2003; Kaya and Öztürk, 2015). If the players do not have the ability to regulate their stress response and their emotions, the stress might be upheld (Gross, 2002, 2008). Therefore, both cognitive processing and emotional regulation are found to be important for international chess tournament players (Fadul and Canlas, 2009). As a result, chess is considered especially mentally and cognitively taxing, and efforts that can optimize chess players’ cognitive and emotional abilities are therefore essential for the performance development of chess players (Barrett, 2002; Wan et al., 2011).

### The Importance of Sleep

An important factor to ensure optimal preparation of athletes under the demanding circumstances of chess is sleep (Goldstein and Walker, 2014; Halson, 2014; O’Donnell et al., 2018; Watson, 2017). Sleep is thought to impact both physical development, quality of life and health in a positive way (Watson, 2017). In addition, sleep has been found to be especially crucial for cognitive functioning (Jones and Harrison, 2001; Walker et al., 2002; Belenky et al., 2003; Jarraya et al., 2014; Nusbaum et al., 2018) and emotional regulation (Sinnerton and Reilly, 1992; Baum et al., 2014; Goldstein and Walker, 2014; Watson, 2017). Research has found that sleep deprivation is associated with cognitive lapses and significantly undermines tasks that require flexible thinking (Rossa et al., 2014; Pallesen et al., 2017) as well as mental state and psychological functioning (Scott et al., 2006). The importance of sleep is mainly grounded in the role sleep has in recovery and adaptive processes in the mind and body (Nédélec et al., 2015; Kellmann et al., 2018).

Sufficient amount of sleep that is required to fulfill the recovery and adaptive needs of the mind and the body varies with age across the life span, and also from person to person (Hirshkowitz et al., 2015). It is generally recommended that young adults (age 18–25) should get 7–9 h of sleep each night (Hirshkowitz et al., 2015). Sleep efficiency, which is the ratio of total sleep time and the total time in bed, of ≥85% is regarded as good sleep quality (Ohayon et al., 2017). Currently, there are no specific recommendations for sleep durations and sleep quality in athletes. Taking into consideration the heavy mental and physiological loads athletes are exposed to, it would be reasonable to expect that athletes’ ideal sleep durations and sleep quality lie within the upper levels of the general recommendations for sleep durations (Watson et al., 2015; Simpson et al., 2017).

### The Sleep Stages

Importantly, it is not just the amount of sleep, but also a suitable distribution of time in each sleep stage that seems to be important for recovery (Rechtschaffen and Kales, 1968; Carley and Farabi, 2016). Sleep is normally divided into rapid eye movement sleep (REM) and non-REM sleep, which is further divided into light and deep sleep. The different sleep stages are distributed into 4–6 cycles throughout the night, each lasting about 60–90 min (Carley and Farabi, 2016).

#### REM Sleep

During REM sleep, the eyes move rapidly from side to side, and brainwave activity and respiration are similar to waking. It is in REM sleep dreaming occurs. As we dream, there is muscle atonia of arms and legs, temporally paralyzing the body in order to prevent the acting out of dreams. The first cycle of REM sleep normally occurs 90 min after falling asleep (Carley and Farabi, 2016), and the prevalence of REM sleep is greater toward the morning hours. REM sleep has been implicated in emotional regulation (Goldstein and Walker, 2014), providing therapeutic depotentiation of emotions from prior experiences and recalibration of noradrenergic salience signaling by the brain, which provides optimal emotion sensitivity and specificity (Goldstein and Walker, 2014). Thus, REM sleep is important for emotional recovery and adaptation. About 20–25% of total sleep during one night is in REM sleep.

#### Non-REM Sleep

Non-REM sleep is divided into three different sleep stages, each associated with specific brain waves and neural activity. Sleep stage 1 and 2 are normally defined as light sleep. In light sleep, brain waves, respiration, and eye movements start to slow down compared to wakefulness. About 50% of total sleep during one night is in the light sleep stage. Stage 3 of non-REM sleep is defined as deep sleep. The proportion of deep sleep is the biggest in the first half of the night. During deep sleep, brain waves, the respiratory system, and muscle activity are at its lowest. Research has found that deep sleep is of great importance for learning and memory consolidation (Aeschbach et al., 2008; Spencer et al., 2017). Typically, about 20–25% of total sleep during one night is in deep sleep.

#### Regular Sleep Patterns

In addition to the recommended sleep durations discussed above and the suggested distribution of the different sleep stages, it is also recommended that the sleep pattern of athletes should be approximately consistent over time. Holding consistent sleep/wake patterns, with regular timings of sleep onset and sleep offset, is one of the most important pillars of good sleep hygiene and adequate sleep quality (Brown et al., 2002). However, results from earlier studies show that athletes have highly variable sleep patterns, especially when comparing weekdays and weekends (Hrozanova et al., 2018).

### Sleep and Chess – The Present Study

Sleep is undoubtedly an important factor to ensure an optimal preparation of chess players. Based on the theoretical introduction in the current study and the claim that chess is a model task environment for research related to psychological processes (Charness, 1992) it is surprising that an earlier study found no associations between chess players’ international and national chess ratings and their perceived sleep quality (Dincel et al., 2015). Based on the importance of sleep on both cognitive processing and emotional regulation, it would be reasonable to expect that such associations do exist. However, to the authors’ knowledge, no studies can be found that confirm these associations.

In general, studies conducted in sports have a long history of focusing on expertise differences (Furley and Memmert, 2012; Jacobson and Matthaeus, 2014; Heppe et al., 2016) as this allows for the investigation of the factors contributing to performance development. In the present study, authors included a longitudinal perspective by following the performances of chess players over a period of 4 months. The aim of the current study was to investigate the associations between performance development and objectively measured sleep among Norwegian chess players. It was hypothesized that the sleep patterns of chess players with differing performance development would vary.

## Materials and Methods

### Participants

Participants were recruited from a Norwegian private school for elite sports where chess is one of the main sports. A total of 15 junior athletes from the chess class at the school and one senior chess athlete were invited to an information meeting about the research project, in which the importance, scope and the data collection process were explained in detail. Athletes who showed interest were instructed to sign up for the study and were given a consent form, approved by the local REC board, and an agreement form for the use of equipment. Athletes who signed and returned these, qualified for participation in the study.

Norwegian schools for elite sports provide an unique opportunity for the development of young athletes who have ambitions to succeed in their sports. Systematic training is a part of athletes’ educational plan. All participating athletes gave their informed consent to participate in the study. REC Central, the Regional Committee for Medical and Health Research Ethics (REC) in Central Norway, founded on the Norwegian law on research ethics and medical research, has approved the study (project ID 2017/2072/REK midt).

### Instruments

The instruments used in this study included the FIDE ratings of the chess players’ performance level, and the Somnofy sleep monitor for sleep measurement. Chess players’ performance levels were retrieved from the FIDE’s home page, where international rating scores are displayed. All international chess competitions are regulated by FIDE and they continually present a rating system of the players. The rating system is based on the games of the players, the rating of their opponents and the results of their games. In the current study, the standard rating (RT) of the players was used. To evaluate how chess players developed during the period of data collection, the RT scores of the players from January the 31st 2019 were compared with their RT scores on the 31st of May 2019.

The Somnofy sleep monitor is a novel, fully unobtrusive tool for sleep assessment, utilizing an impulse radio ultra-wideband (IR-UWB) pulse radar and Doppler technology. The IR-UWB radar emits radio wave pulses in the electromagnetic spectrum, which are able to pass through soft materials (i.e., clothes or duvets), but are reflected by denser materials (i.e., human body). As the pulses are reflected, they are returned and received by the IR-UWB radar again. Then, time-of-flight is used to analyze the time it takes to cover the distance between the radar to the object, and then back to the radar. The movement of the sleeping person and the person’s respiration rate are derived from the IR-UWB radar by utilizing the Doppler effect and Fast Fourier Transform. In this way, Somnofy is able to monitor the vital signs, movement and respiration, of the individual in bed with high precision. The raw data (movement and respiration) from the IR-UWB pulse radar are processed by a sleep algorithm, which uses machine learning to calculate relevant sleep variables. Recently, a full validation of Somnofy against the gold standard in sleep measurement – manually scored polysomnography (PSG) was carried out. The study has shown Somnofy to be an adequate measure of sleep and wake, as well as sleep stages, in a healthy adult population (Toften et al., 2020). For the purposes of this study, the following sleep variables were obtained from the Somnofy sleep monitor: sleep onset, sleep offset, time in bed, sleep onset latency, total sleep time, time and percentage in sleep stages (light, deep and REM), sleep efficiency, and respiration rate (RP). A short description of the sleep variables that were detected in the study is shown in Table 1.

**TABLE 1:** Complete list of sleep variables derived from the sleep algorithm used in the sleep monitor.

Sleep variable	Units	Characteristics of sleep variable
Sleep onset	hh:mm	Time when sleep starts
Sleep offset	hh:mm	Time of wake-up
Time in bed	H	The time spent in bed, including awake time
Sleep onset latency	H	The time it takes from when the athlete intends to go to sleep and actually starts to sleep
Total sleep time	H	Total sleep time obtained from sleep onset to time at wake-up
Light sleep	h/%	Total amount of time / proportion in light sleep (stage N1 and N2)
Deep sleep	h/%	Total amount of time / proportion in deep sleep (stage N3)
REM sleep	h/%	Total amount of time / proportion in REM sleep
Sleep efficiency	%	The percentage of time from sleep onset to wake-up time that was spent asleep
NREM RPM	Number	The number of respiratory ventilations per one minute

*REM, rapid eye movement; NREM RPM, non-rapid eye movement respiration per minute.*

### Procedure

Once all participating athletes returned the signed consent forms, the necessary equipment for sleep monitoring was delivered, along with instructions for correct use. Athletes were instructed on the correct placement of the sleep monitor and the importance of correct settings for optimal functionality. Data collection lasted for 120 days, from the first of February 2019 to the end of May 2019, and entailed day-to-day monitoring of athletes’ sleep patterns. Researchers had access to real-time overview of each participants’ compliance with the study, and monitored the progress closely throughout the whole 120-day period in order to address and solve any technical issues that could occur in relation to the sleep monitoring systems.

The worldwide ranking (FIDE) and the RT scores of the players before the sleep monitoring started were used as a baseline to detect if the players’ ranking scores developed positively or negatively during the sleep monitoring period. The chess players’ baseline RT scores were compared with their scores at the end of the study. Each athlete whose score increased in the period of sleep monitoring was included in the positive performance development group, and each athlete whose score decreased was included in the negative performance development group.

### Inclusion and Exclusion Criteria

The chess players in the current study did all priority chess as the central focus and had all high ambitions regarding their efforts as players. The players’ ambitions and talents are two important inclusion criteria to start at the private school and begin in the class of chess players. As part of their curriculum, chess players trained for 2½ h each day at school. In addition, they trained for at least 10 h each week. During tournaments the time increased. Players spent approximately 6–7 h playing chess each day for 9 days when they competed in tournaments. Tournaments are played during the whole year. Thus, the chess training amounts were at least 22½ h each week.

As the period of the data collection spanned an extensive period of 120 days, some of the sleep data was lost. The main reason for missing data were technical issues when connecting the sleep monitor to the wireless internet network at hotels, when chess players participated in tournaments. An exclusion criterion based on the amount of missing sleep data points per participant was set at maximum 75%.

### Statistical Analyses

The collected data created a clustered data structure, in which repeated measurements of objective sleep data were clustered within 14 individual chess players. By virtue of the clustered data structures, there is dependence of the repeated measurements within individuals. If this dependence is not taken into consideration in the statistical approach, an issue with excessive Type I errors and biased parameter estimates might occur. Therefore, multilevel modeling in Mplus, version 8.3 (Muthén and Muthén, 2017) was utilized to carry out the statistical analyses by clustering the repeated measurements (level 1) within the individual chess players (level 2).

Random intercept models were used to investigate whether sleep parameters varied based on the chess players’ development in their FIDE ranking scores during the period of sleep monitoring. The development in the FIDE RT ranking score is from now defined as the players performance development. Random intercept models assume that the only variation between individuals is at their intercept, and that the effects of the predictor variables are the same for each individual (fixed slope). In the models, the different sleep variables represented the outcome. As the predictor variable (performance development) did not vary on the within-level (athletes had either positive or negative performance development), the random intercept models were modeled only on the between-level. Analyses were separated into weekdays and weekends to investigate whether the chess players held consistent sleep/wake patterns. For each random intercept model, intra-class correlation (ICC), or the extent the dependent values of occasions of measurement in the same participant resemble each other as compared to those from different participants was calculated. IBM SPSS (version 25.0) was used to conduct demographic analyses and descriptive statistics.

## Results

Fifteen high school athletes and one senior athlete returned the signed consent forms and were enrolled in the study. Of these, two athletes dropped out of the study. Thus, 14 athletes (13 males and 1 female, mean age 21.8 ± SD 8.7, range 17–52 years) completed the study and as a result, 1680 data points with sleep could be collected. For objective sleep data, 1009 or 60%, of the potential 1680 nights of sleep data were collected and analyzed. Data was mainly lost due to the athletes dropping out, technical issues with connecting the Somnofy units to Wi-Fi, especially at tournaments where athletes needed to stay at hotels with a locked Wi-Fi solution, and travels where athletes were unable to use the sleep monitoring device.

### Descriptive Statistics

The mean RT score was 2242 on January 31st, 2019 (std = 177.5, min = 1822, and max = 2576), and 2260 on May 31st, 2019 (std = 168, min = 1809, and max = 2569). The chess players RT scores before the sleep monitoring started was used as a baseline to investigate if the score increased, decreased or was stable throughout the period. The RT score of the world’s number one chess player is currently 2872. Descriptive statistics of the chess players’ sleep patterns are shown in Table 2.

**TABLE 2:** Descriptive statistics for the objective sleep patterns, based on 1009 nights of data in 14 chess players.

Sleep variable	Mean	STD
Sleep onset (hh:mm)	00:58	01:56
Sleep offset (hh:mm)	08:25	02:12
Time in bed (h)	09:31	03:00
Sleep onset latency (h)	00:39	00:42
Total sleep time (h)	06:44	01:35
Light sleep (h)/(%)	03:49/56.4	01:00/8.1
Deep sleep (h)/(%)	01:16/19.3	00:25/6.2
REM sleep (h)/(%)	01:39/23.8	00:40/6.5
Sleep efficiency (%)	72.8	14.7
NREM RPM (N)	14.0	2.4

*STD, standard deviation; REM, rapid eye movement; NREM RPM, non-rapid eye movement respiration per minute.*

### Performance Development and Sleep Patterns

The comparison of RT January with RT May showed that the mean development in RT was 17 (std = 71, min = −153, and max = 184). Seven of the chess players had negative development in their RT scores (P− group), while seven had positive development (P+ group). Thus, two groups were included for further analysis, the P+ and P− group. The P− group had a mean RT score of 2232 at May 31st (std = 220, min = 1809, and max = 2569), whereas the P+ group had a mean RT score of 2288 (std = 81, min = 2118, and max = 2376). Since a stable sleep pattern across time was found to be important (Hrozanova et al., 2018) the analysis in the current study was separated into weekdays and weekends. Table 3 shows the sleep variables in the two groups during weekdays, while Table 4 shows the sleep variables in the two groups during weekends.

**TABLE 3:** Two-level regressions with performance development (0 = negative, *N* = 7; 1 = positive, *N* = 7) as IV and weekday sleep variables as DV.

DV	ICC	Est. P−	S.E. P−	Est. P+	S.E. P+	ΔP	Sig.	*R*^2^ (%)
Sleep onset (hh:mm)	0.57	00:47	0:30	01:25	0:43	0:38	0.378	5.4
Sleep offset (hh:mm)	0.57	07:51	0:26	08:54	0:49	1:03	0.194	10.9
Time in bed (h)	0.39	09:00	0:29	10:10	1:01	1:10	0.253	8.9
Sleep onset latency (h)	0.54	00:34	0:07	00:58	0:17	0:24	0.149	13.2
Total sleep time (h)	0.32	06:25	0:17	06:47	0:29	0:20	0.471	3.7
Light sleep (h/%)	0.29/0.23	3:37/55:43	0:08/1:32	3:52/56:41	0:18/2:10	0:15/0:58	0.394/0.653	5.2/1.5
Deep sleep (h/%)	0.31/0.26	1:13/19:11	0:06/1:23	1:21/20:29	0:07/1:45	0:08/1:18	0.265/0.452	8.6/4.1
REM sleep (h/%)	0.29/0.32	1:37/24:03	0:08/1:23	1:34/22:37	0:12/1:59	−0:02/−1:26	0.870/0.470	0.2/3.7
Sleep efficiency (%)	0.48	72.91	2.91	68.94	5.34	−3.97	0.457	3.9
NREM RPM	0.91	15.51	0.67	12.67	1.02	−2.83	*0.005*	35.8

*IV, independent variable; DV, dependent variable; ICC, intraclass correlation; Est. P−, estimate negative performance group; S.E. P−, standard error negative performance group; Est. P+, estimate positive performance group; S.E. P+, standard error positive performance group; ΔP, change in performance group estimate; R^2^, explained variance; REM, rapid eye movement; NREM RPM, non-rapid eye movement respiration per minute. Regressions were modeled on the between-level, and clustered on participant. Values are unstandardized and significant results are italicized.*

**TABLE 4:** Two-level regressions with performance development (0 = negative, *N* = 7; 1 = positive, *N* = 7) as IV and weekend sleep variables as DV.

DV	ICC	Est. P−	S.E. P−	Est. P+	S.E. P+	ΔP	Sig.	*R*^2^ (%)
Sleep onset (hh:mm)	0.41	01:15	0:25	02:15	0:43	1:01	0.156	13.8
Sleep offset (hh:mm)	0.46	09:09	0:36	09:47	0:54	0:38	0.482	3.7
Time in bed (h)	0.34	09:40	0:49	09:53	1:00	0:13	0.835	0.3
Sleep onset latency (h)	0.32	00:29	0:07	00:47	0:12	0:17	0.141	15.6
Total sleep time (h)	0.19	06:49	0:20	06:47	0:25	−0:02	0.944	0.0
Light sleep (h/%)	0.16/0.18	3:53/56:41	0:10/0:53	3:55/57:37	0:15/2:07	0:03/0:56	0.904/0.659	0.1/1.8
Deep sleep (h/%)	0.32/0.25	1:11/17:26	0:05/1:03	1:20/20:15	0:08/1:32	0:08/\2:49	0.273/0.066	9.0/2.3
REM sleep (h/%)	0.22/0.26	1:45/25:09	0:08/1:11	1:33/22:08	0:10/1:42	−0:12/−3:00	0.241/0.077	10.9/21.5
Sleep efficiency (%)	0.37	72.97	3.26	70.04	4.91	−2.93	0.552	2.7
NREM RPM	0.84	15.51	0.60	12.91	0.98	−2.60	*0.008*	33.9

*IV, independent variable; DV, dependent variable; ICC, intraclass correlation; Est. P−, estimate negative performance group; S.E. P−, standard error negative performance group; Est. P+, estimate positive performance group; S.E. P+, standard error positive performance group; ΔP, change in performance group estimate; R^2^ = explained variance, REM, rapid eye movement; NREM RPM, non-rapid eye movement respiration per minute. Regressions were modeled on the between-level, and clustered on participant. Values are unstandardized and significant results are italicized.*

The analysis comparing sleep patterns during weekdays in the P+ and the P− groups showed that sleep patterns were different across the two groups, although the results did not reach significance. The P+ group fell asleep at 01:25 on average and woke up at 08:54, whereas the P− group fell asleep at 00:47 and woke up at 07:51. Thus, the P+ group also fell asleep later at night and woke up later in the morning compared with the P− group. The sleep offset variable explained 10.9% of the variance in the model. Sleep onset latency was longer by 24 min for the P+ group compared to the P− group and this variable explained 13.2% of the variance in the model. The P+ group had on average 22 min longer sleep per night and time in bed explained 8.9% of the variance in the model. The P+ group also had longer deep sleep than the P− group (by 8 min), which explained 8.6% of the variance in the model. The P+ group also spent more time in light sleep (by 15 min) than the P− group. The P− group had more time in REM sleep (by 3 min), than the P+ group. The P+ group spent 1 h and 10 min more time in bed compared to the P− group. The P− group also had better sleep efficiency compared to the P+ group, by 4%. The respiration rate during non-REM sleep was the only variable that reached significance, whereas the P− group measured 2.84 respiratory ventilations more per minute compared to the P+ group. NREM RPM explained 35.8% of the variance in the model. The ICC ranged from 23 to 57% for the sleep variables, denoting that repeated measurements of sleep variables were 23–57% similar within groups. The rest of the variation may be attributed to between-level factors. NREM RPM showed a high ICC of 91%, indicating that observations within a cluster are highly similar.

The P+ group fell asleep at 02:15 at weekends and woke up at 09:47, whereas the P− group fell asleep at 01:15 and woke up at 09:09. Thus, the pattern was consistent with the results from weekdays where the P+ group fell asleep later at night and woke up later in the morning compared to the P− group. However, the sleep pattern was delayed with almost 1 h. The sleep onset variable explained 13.8% of the variance in the model. The results from weekends further show that total sleep time was almost similar between the groups and that the amount of time in REM sleep was 12 min longer in the P− group. REM sleep explained 10.9% of the variance in the model. The time in light sleep was almost similar, where the P+ group spent 2 min longer in light sleep. The time in deep sleep was consistent with the results for weekdays and the variable explained 9% of the variance in the model. The respiration rate during non-REM sleep was the only variable that reached significance at weekends, whereas the P− group measured 2.6 respiratory ventilations more per minute compared to the P+ group. NREM RPM explained 33.9% of the variance in the model. The ICC ranged from 16 to 46% for the sleep variables, denoting that repeated measurements of sleep variables were 16–46% similar within groups. The rest of the variation may be attributed to between-level factors. NREM RPM showed a high ICC of 84%, indicating that observations within a cluster are highly similar.

## Discussion

The current study investigated the associations between objectively measured sleep patterns and performance development among Norwegian chess players over a period of 4 months. It was hypothesized that sleep patterns among chess players with differing performance development would vary. A comparison of the standard rating scores (RT) retrieved from the world ranking list on the FIDE’s homepage showed that seven of the chess players had positive performance development (P+) and seven had negative performance development (P-). Based on the FIDE’s world ranking RT scores one group was defined as P+ (positive performance development group) and P− (negative performance development group). The results in the current study confirm the hypothesis, showing differences in sleep patterns in the two groups, suggesting that sleep is associated with performance development in the current sample of chess players. However, the respiration rate during non-REM sleep was the only variable that reached significance on both weekdays and weekends. The present results should be interpreted with the low number of participants in the two groups in mind. This is a major limitation of the present study. Low number of participants in each group influences the power to find significant differences in multilevel statistical analyses.

### A Minimum of the Recommended Sleep

The total amount of sleep detected in both groups was well below, or in the lower limit, of the sleep recommendations introduced by Hirshkowitz et al. (2015). Currently, there is no consensus in the literature on the amount of sleep athletes are obtaining. A recent review paper on sleep in athletes has shown similar sleep durations (O’Donnell et al., 2018) while another study in junior athletes has shown significantly longer sleep durations (Hrozanova et al., 2018). Furthermore, the sleep/wake times detected in the current study show that sleep starts later at night and ends later in the morning than what was found among junior athletes in earlier studies (Hrozanova et al., 2018). Future research should investigate the possible role played by the extent of blue light chess players are exposed to. It is possible that chess players spend more time on blue light-emitting devices than other groups of junior athletes, which may negatively influence their sleep patterns.

The current study did not find any significant differences in total sleep time between the two groups, but the P+ group had on average 22 min longer sleep time than the P− group and spent 1 h and 10 min more time in bed during weekdays. However, the results also showed that it took the P+ group longer to initiate sleep, compared to the P− group. This result indicates that the P+ group intended to sleep more, or at least that they intend to fall asleep earlier at night. Such intention might also be the reason for the difference between the groups in sleep onset. At weekends these differences were almost balanced.

The sleep efficiencies in the current study (mean 73%) were also below the recommended levels (>85%). In addition, sleep efficiency varied between the P+ and the P− group during weekdays by 4% and weekends by 2.9%, but this did not reach significance. Importantly, research claims that extending sleep duration beyond the recommended duration has been associated with improved athletic performance (Mah et al., 2011). Likewise, improved sleep efficiency is likely to improve functioning during the daytime (Kirmil-Gray et al., 1984). Therefore, the current results give reason to question whether the chess players in both groups receive the required amount and quality of sleep that they need to adapt and recover from their daily loads.

### REM Sleep and Emotional Regulation

The current study also found differences in the amount of REM sleep between the groups. The P− group spent more time in REM sleep than the P+ group, especially at weekends where the variable explained 10.9% of the variance in the model. Interestingly, the minute respiration (RP) was the only significant variable and was significantly higher in the P− group, both during weekdays and weekends. The RP explained 35.8 and 33.9% of the variance in the model. Research claims that when humans are faced with internal or external stressors, the respiratory ventilation will increase (Tipton et al., 2017). Following the stress argument, the REM stage is essential for emotional regulation. More time in REM sleep might indicate a higher need for emotional regulation in the P− group compared to the P+ group. Thus, an increase in the number of breaths per minute (respiratory frequency) might indicate more internal or external stress.

Importantly, stress is considered the most common predictor of sleep disturbance and is therefore likely to have a detrimental impact on the optimal preparation of chess players (Morin et al., 2003). The association between REM sleep and performance development may be due to the fact that the P+ group was experiencing more eustress due to their upward development trajectories, while the P− group might have been experiencing more distress due to their downward performance development throughout the duration of the study (Fuentes et al., 2018). It is therefore reasonable to assume that the need for emotional regulation and REM sleep in the two groups was different. Accordingly, the percent amount of time that the P− group spent in REM sleep was in the upper limit of what is recommended, which might indicate a higher daytime stress arousal in need of emotion regulation during the sleep period. This might serve as a potential explanation for the differences in REM sleep between the P− group and P+ group.

In line with the proposed mechanisms at play, research has found that higher stress levels during the day were associated with higher cognitive and somatic arousal at bedtime, and that the bedtime arousal influenced both sleep efficiency and sleep quality negatively (Morin et al., 2003). Furthermore, research has found that when players are exposed to stress, their attention tends to focus inward, toward thoughts and emotions (Wells and Matthews, 1994; Wells, 2009) and that stress has a negative influence on their attentional control (Eysenck et al., 2007). Thus, the P− group might have been exposed to more underlying negative thoughts and emotions at bedtime because of their negative performance development (Morin et al., 2003). However, we cannot draw causal conclusions from these results, and future research needs to investigate these potential associations.

### Deep Sleep and Learning

There were also differences in the time spent in deep sleep between the P+ and P− groups and the variable explained, respectively, 8.6 and 9% in weekdays and weekends of the variance in the model. The P+ group spent more time in deep sleep than the P− group both in weekdays and weekends. Since recent research has shown that deep sleep is essential for learning and memory consolidation (Spencer et al., 2017) a possible explanation for the current findings might be that the obtained amount of deep sleep is a potential key for performance development among the chess players. The acquired neurological patterns that are required to understand and perform the game of chess on expertise levels are not solely a result of innate abilities, but also a result of deliberate practice, learning and memory consolidation (Ericsson and Staszewski, 1989; Ericsson and Harris, 1990; Ericsson and Kintsch, 1995; Charness et al., 2005). Each game of chess has numerous alternative chess patterns, combinations and moves. On deciding the best move, memorization of all the possible alternatives is key. Expert chess players have been found to have superior memory for chess positions and problem solving (Chase and Simon, 1973a, b; Gobet et al., 2004) better abilities to extract chess relations in parallel (Charness et al., 2001), attentional capacity and control (De Groot and Gobet, 1996; Reingold et al., 2001) and a richer network of chess patterns in long-term memory structures (Ericsson and Kintsch, 1995). Thus, learning and development of these cognitive functions are essential to perform at the highest levels in chess and acquired amounts of deep sleep may be therefore essential for optimization of performance development among chess players.

Interestingly, the results also showed that the P− group obtained deep sleep below the recommended levels (19.2% in weekdays, 17.4% in weekends). Given the importance of learning and memory consolidation in chess, the amount of deep sleep in this group may be of importance. However, even though the P+ group obtained higher amounts of deep sleep (20.5% in weekdays, 20.3% in weekends), these amounts are still in the lower limit of what is recommended. These results give reason to question the extent to which deep sleep influences the performance development of athletes, and whether the P− group could benefit from higher amounts of deep sleep. Unfortunately, we cannot draw conclusions about this potential association and future research needs to investigate this to conclude.

### Conclusion and Limitations

To the authors’ knowledge, this is the first study that uses an objective validated measurement of sleep over a long period of time, that shows associations between sleep and performance development among chess players. Results of the multilevel analyses in the current study showed that the sleep patterns of the chess players with positive performance development were different from the group of players with negative performance development. Due to low sample size, the measured sleep variables did not reach significance. Differences between the groups, albeit insignificant, were nevertheless found: the P+ group obtained higher amounts of deep sleep, less REM sleep and had significantly lower respiration rate compared to the P− group. These results give reason to discuss whether the differences in sleep patterns have consequences for the learning and memory consolidation of the chess players, and whether the P− group experienced more internal or external stress compared to the P+ group, which would again influence their sleep negatively. The results also indicate that both groups can obtain better recovery and adaptation from higher amounts of total sleep and improved sleep efficiencies.

Even though multiple interesting results are presented in this study, several limitations should be kept in mind when interpreting the results. First of all, 40% of all potential data was lost mainly because of difficulties with the manual pairing of the sleep monitor to a local Wi-Fi network, especially when the players competed in tournaments and stayed at different hotels with a closed Wi-Fi solution or a bad Wi-Fi connection. Secondly, the number of participants was low and influenced the power of the multilevel approach used in the current study. This is likely the main reason why some of the findings, despite considerable differences between groups, failed to reach significance. Thirdly, there are other relevant variables that may influence players’ sleep patterns, that were not considered in the present study – for instance players’ training loads and daily stress loads. Future research should collect data on these variables to control for their effects. Finally, the study did not control for sleep during daytime and napping frequency (Petit et al., 2014) and interpretation of the results must take that into consideration. Future studies should also consider detecting daytime sleep and napping.

## Data Availability Statement

The datasets generated for this study are available on request to the corresponding author.

## Ethics Statement

REC Central, the Regional Committee for Medical and Health Research Ethics (REC) in Central Norway, founded on the Norwegian Law on Research Ethics and Medical Research, has approved the study (Project ID 2017/2072/REK midt). The participants provided their written informed consent to participate in this study.

## Author Contributions

FM and MH contributed to the conception and design of the study, performed the statistical analysis, and wrote the first draft of the manuscript. MO, FM, and MH organized the database. MO wrote sections of the manuscript. All authors contributed to the manuscript revision, read, and approved the submitted version.

## Conflict of Interest

The authors declare that the research was conducted in the absence of any commercial or financial relationships that could be construed as a potential conflict of interest.
